# CO_2_ and electrical stunning differentially affect energy metabolism in pigs

**DOI:** 10.1038/s41598-025-10874-6

**Published:** 2025-07-17

**Authors:** Manuela Peukert, Sebastian Zimmermann, Björn Egert, Dagmar Adeline Brüggemann

**Affiliations:** 1 Department of Safety and Quality of Meat, Max Rubner-Institut, 95326 Kulmbach, Germany; 2 Department of Quality and Safety of Fruit and Vegetables, Max Rubner-Institut, 76131 Karlsruhe, Germany

**Keywords:** Electrical stunning, CO_2_ stunning, Hypoxia, Metabolomics, Energy metabolism, Metabolism, Metabolomics

## Abstract

The use of CO₂ or electrical stunning to render pigs unconscious and insensible before exsanguination is a common practice. Although both methods are widely implemented, they differ fundamentally in their underlying mechanisms, and studies have produced conflicting results regarding their influence on meat quality. In the case of CO₂ stunning, impaired pulmonary gas exchange causes a rapid onset of systemic hypoxia, accompanied by hypercapnia, which in turn lowers blood pH and triggers additional physiological stress responses. Electrical stunning, by contrast, induces a generalized epileptic seizure by depolarizing neuronal cell membranes, markedly increasing oxygen consumption in both the brain and peripheral muscles. Coupled with apnea-induced cessation of respiration, this leads to rapid systemic hypoxia and associated metabolic disturbances. With this study, we aim to generate comprehensive metabolic data as a foundation for deeper understanding of the animal’s physiological responses depending on the stunning method used. We identified differences in metabolite pathways associated with the stunning method and evaluated potential influence on early *post mortem* processes relevant to meat quality development. After CO_2_ stunning, there was a marked increase in purine degradation into inosine (*p* < 0.0001) and hypoxanthine (*p* < 0.0001), along with increased levels of C4 intermediates (succinate, fumarate and malate, all *p* < 0.0001) in the tricarboxylic acid (TCA) cycle. In contrast, electrical stunning showed a higher rate of glycolysis, as indicated by reduced levels of C6 sugars (e.g. glucose *p* < 0.0001), and elevated levels of TCA cycle entry metabolites such as citrate (*p* = 0.0053) and aconitate (*p* = 0.0009). Our findings suggest that purinergic signaling acts as a rapid emergency response mechanism during gas stunning, reflected by pronounced purine catabolism. The distinct metabolite patterns likely result from different physiological stress responses, such as CO_2_-induced acidosis and variable oxygen availability. In addition, differences in cellular redox balance (NAD⁺/NADH) between stunning methods may further modulate glycolytic flux and TCA cycle activity. These divergent metabolic states at the time of death may, in turn, influence subsequent *post mortem* biochemical processes and ultimately influence meat quality development.

**Supplementary Information:**

The online version contains supplementary material available at 10.1038/s41598-025-10874-6.

## Introduction

The stunning process at slaughter is intended to induce unconsciousness and insensibility prior to exsanguination. In commercial European slaughterhouses, pigs are typically stunned either electrically or by exposure to a high CO_2_ concentration. During electrical stunning, a defined current (minimum of 1.3 A for at least 4 s) spans the brain^1^, causing strong depolarization of nervous cell membranes. This results in excessive, uncoordinated activity resembling an epileptic seizure^2,3^. Additional cardiac perfusion induces irreversible ventricular fibrillation. The excessive increase in activity during these epileptic-like seizures leads to rapid shifts in energy and oxygen demand. Combined with respiratory arrest and tachycardia, a hypoxic environment is established throughout the body. As aerobic metabolism decreases under these conditions, glycolysis is accelerated, leading to increased lactic acid production. In addition to the rapid metabolic changes, blood pressure increases, which can cause rupture of blood vessels and hemorrhages. Nass et al.^4^ reported similar phenomena in epileptic patients, noting increased lactic acid, creatinine and ammonia levels during tonic–clonic seizures.

In contrast, gas stunning with CO₂ relies on the narcotic and respiratory-stimulating effects of carbon dioxide. Typically, a concentration of > 80% in a deep pit is used. Following absorption into the bloodstream, CO₂ crosses the blood–brain barrier and diffuses into the cerebrospinal fluid, leading to cerebral acidosis. This altered brain environment impairs central nervous system function and subsequent loss of consciousness. Notably, blood pH decreases significantly from 7.3–7.5 to 6.6–6.9 during CO₂ stunning, as reported by Martoft^6^ and Hognestad^5^. Additionally, Martoft et al.^6^ also observed an intracellular pH decrease in the brain to pH 6.7 within 60 s exposure to 90% CO2. In comparison, electrical stunning causes a less pronounced pH reduction, with Becerril-Herrera^7^ documenting a decrease to pH 7.14. The duration of exposure in the CO₂ pit ranges between 100 and 180 s, which is longer than the process of electrical stunning. During the initial 10–40 s, animals often exhibit aversive reactions such as increased activity and vocalizations, indicating a period of distress^8,9^.

While CO₂ stunning enables group handling of pigs and is associated with advantages in terms of throughput and operator safety, it remains controversial due to persistent animal welfare concerns. In contrast, electrical stunning is considered to induce unconsciousness more rapidly, but requires individual animal restraint and precise electrode placement, which can itself be stressful for the animals. Animal welfare during stunning is a major focus of current research and public debate. However, in addition to minimizing animal suffering, stunning methods must also preserve meat quality. It is well established that stress experienced by pigs shortly before or during slaughter can negatively impact meat quality^10–13^. This is closely linked to disruptions in energy metabolism, as a balanced energy status is a prerequisite for optimal meat characteristics such as pH, water-holding capacity, and color. Acute stress leads to imbalances in energy metabolism, which in turn can compromise the conversion of muscle to meat. Therefore, understanding how different stunning methods influence metabolic pathways—particularly those associated with energy metabolism—is essential. Such knowledge can contribute to the development of slaughter practices that are both ethically sound and capable of maintaining high product quality. This study was based on the hypothesis that CO₂ and electrical stunning induce distinct metabolic responses in pigs, resulting in divergent metabolite profiles, particularly in pathways linked to energy metabolism and meat quality. To elucidate these fundamental metabolic differences and connect them to relevant biochemical pathways, a nontargeted metabolite profiling approach was applied, complemented by targeted quantification of purines and TCA cycle metabolites in sticking blood.

## Methods

The study aimed at the investigation of metabolic pathway differences upon two stunning techniques in pigs—CO_2_ and electrical stunning. Therefore, metabolites were analysed by nontargeted two-dimensional gas chromatography coupled mass spectrometry (GCxGC-qMS) profiling, and quantification of purines and tricarboxylic acid cycle (TCA) metabolites.

### Animals and sample acquisition

Blood samples were randomly collected at 1 day from 35 finishing pigs (gilts and surgically castrated males at a ratio 1:1, slaughter weight 110–120 kg) at a commercial slaughterhouse. No ethical approval was required for this study because the blood samples were collected during the routine slaughter process at a commercial slaughterhouse. All animals were stunned using standard procedures (either electrical or CO₂ stunning) prior to bleeding, in accordance with applicable animal welfare regulations. The sampling did not involve any additional handling or interventions beyond the conventional slaughter process. The animals originated from a single herd, ensuring uniformity in feeding and age. Prior to exsanguination, the pigs were alternately subjected to either electrical stunning or carbon dioxide (CO₂) stunning, in accordance with standard commercial slaughter practices. For CO_2_ stunning, a concentration of 95% CO_2_ in a dip-lift system was used at a time interval of 100 s. The time between CO_2_ stunning and exsanguination was ca. 20–30 s so that all the observed metabolic shifts were induced within 120 s. Electrical stunning was carried out using a hand-held stunning device (Haas, Neuler, Germany) by head stunning for 6 s followed by cardiac arrest stunning for 4 s, each at 230 V and 1.6 Ampere. The time interval between stunning and bleeding was again about 20–30 s, resulting in an overall time from the start of stunning to bleeding of approximately 50 s. Blood samples were collected during exsanguination, immediately frozen in liquid nitrogen, and stored at − 80 °C until further use.

### Metabolite profiling

#### Chemicals

All the chemicals used were commercially obtained via Merck (Darmstadt, Germany), Altmann Analytics (Munich, Germany) or VWR (Darmstadt, Germany) and are listed in Supplementary Table S1.

#### GCxGC-qMS metabolite analyses

Sample preparation for GCxGC-qMS metabolite profiling was performed according to Wagner et al.^14^. In brief, frozen blood samples were lyophilized and homogenized to a fine powder. A total of 20 mg of the powder was extracted with 600 µl of ice-cold methanol:water (8:2, v/v) containing an internal standard mixture (Supplementary Table S1) using a bead mill homogenizer (Bead Ruptor 24 Elite, Omni International, USA). After centrifugation, the pellet was re-extracted with 600 µl of ice-cold methanol:chloroform (2:1 v/v) following the same procedure as the first extraction. The combined supernatants were transferred into 2 ml glass vials containing 200 µl glass inserts, then dried in a vacuum centrifuge (Christ Speedvac RVC 2–18 CD plus, Germany). The dried extracts were stored under a protective argon atmosphere at − 80 °C until analysis. Quality control (QC) and blank samples were included in the analysis alongside the biological samples. The QC samples were prepared by pooling aliquots from multiple blood samples. The blank samples contained no biological matrix. Prior to GC–MS measurement, the samples were derivatized. First, methoximation was carried out using a 20 mg/ml solution of methoxyamine hydrochloride (MAH) in pyridine incubated at 50 °C for 1 h with shaking. In the second step, 70 µl of N-methyl-N-(trimethylsilyl)trifluoroacetamide (MSTFA) containing 1% trimethylchlorosilane (TMCS) was added, and the samples were shaken at 70 °C for 1 h. The measurements were carried out using a Shimdazu GCMS QP2020 instrument (Shimadzu, Duisburg, Germany). Instrumentation details and parameter setting are provided in Supplementary Table S2. Full-scan data were acquired in a mass range of 60–550 m/z. For nontargeted GCxGX-qMS measurements, total ion chromatogram (TIC) integration was performed using the GCMS Postrun Analysis Module within the instrument software GCMSsolution (Version 4.45, Shimadzu, Duisburg, Germany). Data processing steps included peak filtering, peak alignment, signal intensity drift correction and quality assessment, as described by Egert et al.^15^ and Weinert et al.^16^. Missing values (corresponding to signals that were not detected) were imputed with random values between half the limit of detection (LOD) and LOD. The LOD was estimated as the smallest detected peak area across the entire data set. Compound annotation was performed using the NIST 14 library database, as well as an in-house created library, both of which were implemented in the GCMS Postrun Analysis Module within the instrument software GCMSsolution. Visualization of two-dimensional (2D) chromatograms was done using the GC Image Software (Version 2.7, GC Image, Lincoln, Nebraska). A series of n-alkanes (C7-C30) was used as a retention time standard.

For the analysis of citrate cycle metabolites, the first extraction step was identical to that of nontargeted metabolite extraction. Re-extraction was performed with methanol:water (8:2, v/v). The combined extracts were then divided into two portions: one for direct dry in a measurement vial using a vacuum centrifuge, and the other for a reduction step using borodeuteride. For the reduction, 500 µl of the extract were mixed with 5 µl of 2 N NaOH and 25 µl of freshly prepared 10 mg/ml NaBD_4_ in 50 mM NaOH. The mixture was incubated for 1 h at room temperature with shaking. After incubation, the pH was adjusted to pH 4 with a 3.5 N HCl solution. A 100 µl aliquot was then transferred into a measurement vial and dried in a vacuum centrifuge. A standard curve was prepared in the same manner, incorporating 5 mg of blood matrix to account for matrix effects during MS measurement. The standards and concentrations used are listed in Supplementary Table S1. Prior to GC‒MS measurements, the samples were derivatized, at first using 30 µl of a MAH solution for 1 h at 50 °C, and subsequently with 50 µl of MTBSTFA/TBDMCS for 1 h at 70 °C. The protocol and corresponding calculations were adapted from Mamer et al.^17^. The peak areas for quantifying and qualifying ions were integrated via the implemented Postrun Analysis Module within the instrument software GCMSsolution (Version 4.45, Shimadzu, Duisburg, Germany).

#### Purine quantification by LC-ESI-QToF MS

The blood powder was extracted in two steps using a methanol:water (8:2, v/v) solution, with a final sample-to-volume ratio of 1:8 (w/v). To clean up the raw extract, solid-phase extraction (SPE) was performed using a Waters HLB Prime cartridge (1 cc, 30 mg; Waters, Eschborn, Germany). The flow-through was used for LC-ESI‒QToF MS measurements. For quantification, a calibration curve was prepared and the calibrants were spiked with a quality control sample extract to account for matrix effects. Standards and concentrations used are listed in Supplementary Table S1, and instrumentation details and parameter settings are provided in Supplementary Table S2. Peak areas of the corresponding ions were integrated using the Data Analysis software (V 4.2, Waters, Eschborn, Germany). The ion extraction accuracy was set to ± 0.005 m/z. Additional information on compound integration can be found in Supplementary Table S1.

### Analysis of heat shock protein (HSP) gene expression

Total RNA was isolated from 400 µl EDTA stabilized blood using 1200 µl of Lysis Blood buffer (Roboklon, Germany) following the manufacturer’s instructions. 5 µl of each sample were converted into cDNA using the NG dART RT KIT (Roboklon, Germany) according to the manufacturer’s instructions, and stored at 4 °C until further use. The qPCR was performed on a Bio-Rad qPCR Cycler CFX 96 (Biorad, Germany). For each reaction, 10 µl cDNA was mixed with 2 × SG qPCR Master Mix (Roboklon, Germany), primers (10 pmol/µl of downstream and upstream primer, respectively), and RNA free water to a final volume of 25 µl. The thermal profile was set according to the manufacturer’s guidelines: an initial enzyme activation step at 95 °C for 10 min, followed by 41 cycles of denaturation at 95 °C for 15 s, annealing at 59 °C for 30 s, and elongation at 72 °C for 30 s. A final inactivation step was included at 95 °C for 15 s. Each qPCR run included a negative control (without DNA) alongside the experimental samples. The primers used are described in Zimmermann and Brüggemann^18^. Delta Ct -values were calculated by subtracting the animal-specific Ct values of the housekeeping genes (GAPDH, ß-Actin) from the Ct values of the heat shock proteins. These delta Ct values were used for all further analyses.

### Statistical data analysis

Multivariate and univariate statistical analyses were using the JMP software (17.1.0, SAS Institute Inc., Cary/NC, USA). We applied principal component analysis (PCA) for an overall observation of variations among sample groups. For direct comparison of individual signals, we first conducted a distribution analysis by applying the Anderson Darling test, followed by ANOVA and Wilcoxon tests. The Volcano Plot was generated using R (4.5.0., The R-Project for Statistical Computing), highlighting metabolites with significant fold changes and adjusted p-values (Welch test).

## Results

### Blood metabolite profiles differ as a consequence of the stunning method

**Fig. 1:**
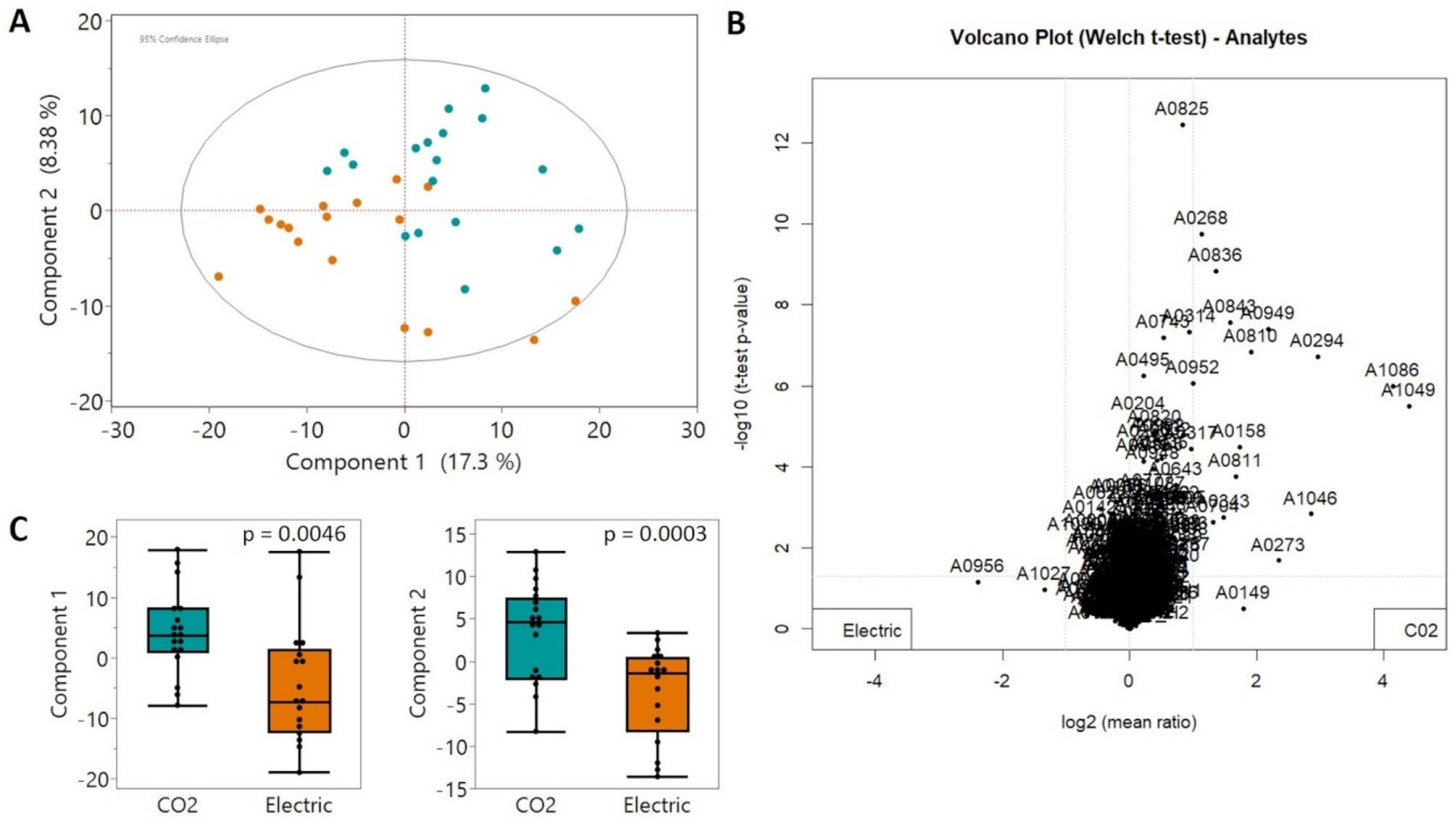
Principal component analysis from nontargeted GCxGC-qMS metabolite profiling of sticking blood from 35 pigs undergoing different stunning methods. (**A**) PCA score plot (**B**) Volcano plot highlighting most significant molecular features (**C**) ANOVA of principle components 1 and 2 indicating a significant difference of metabolite profiles between CO_2_ and electrical stunning. Orange—electrical stunning and turquoise—CO_2_ stunning.

A comprehensive GCxGC-qMS approach was employed to investigate metabolite pathway differences resulting from the stunning method. A total of 513 molecular features were analyzed for statistical evaluation. The nontargeted principal component analysis (PCA), considering all 513 signals, revealed notable metabolic variation both among individual pigs and between stunning techniques (Fig. 1 A). Several variables contributed strongly to this separation, as reflected by their log₂ fold changes shown in Fig. 1B. Principal components 1 and 2 differed significantly between the two stunning methods (Fig. 1C), underscoring the distinct metabolic effects of the treatments.

We applied univariate statistical tests to identify discriminating variables between both stunning techniques. The ANOVA/Wilcoxon tests (*p* ≤ 0.05) revealed 117 variables as significantly different (Supplementary Table S3). For 54 of the diversifying signals, the database search revealed annotations to particular metabolites (level 2) or metabolite groups (level 3) according to the Metabolomics Standards Initiative^19^. The results for the annotated and significant features presented in Table 1 indicate large differences in central metabolite pathways between the stunning techniques.

**Table 1:** Annotated Metabolites from GC–MS analysis showing significant difference between CO_2_ and electrically stunned animals.

Metabolite class	Signal-ID profiling	Annotation	Anderson–darling	ANOVA	Wilcoxon test	% amount CO_2_ stunning to electrical stunning (= 100%)
Glycolysis	A1048	Disaccharide	0.7110	0.0061	0.0087	107.7
	A0919	Glucose	0.9020	< 0.0001	0.0002	116.9
	A1060	Hexosephosphate	0.0180	0.0078	0.0306	146.9
	A0810	GA3P	0.0030	< 0.0001	< 0.0001	378.6
Pentose-Phosphate-Pathway	A0952	Gluconic acid	0.0250	< 0.0001	< 0.0001	200.3
	A0734	Pentose 1	0.2310	0.0208	0.0238	110.7
	A0743	Pentose 2	0.9110	< 0.0001	< 0.0001	145.4
	A0811	Ribonic acid	< 0.0001	0.0001	0.0003	320.1
Other sugars and derivatives	A0805	2-Desoxy-pentose	0.0020	0.0014	0.0007	173.9
	A0814	1,5-Anhydrohexitol 1	0.0020	0.0201	0.0535	125.1
	A0873	1,5-Anhydrohexitol 2	0.2330	0.0337	0.0306	120.1
	A0899	sugar derivative	0.3140	0.0028	0.0059	139.1
	A0907	Inositol	0.6000	0.0312	0.0721	130.8
	A0941	sugar derivative	0.2410	0.0326	0.0153	81.0
	A0948	sugar derivative	0.6070	0.0001	0.0003	129.7
	A0955	sugar derivative	0.1250	< 0.0001	0.0002	135.6
	A0962	Hexose	0.0950	0.0011	0.0012	160.5
	A0982	Methyl galactoside	0.5130	< 0.0001	< 0.0001	136.2
	A1000	Hexose	< 0.0001	0.001	< 0.0001	168.4
	A0273	Aceton	< 0.0001	0.02	0.0259	512.3
TCA cylce	quantitative analysis	Citrate	0.0132	0.0053	0.0116	75.82
		Aconitate	0.0292	0.0009	0.0031	71.86
		Succinate	0.2428	< 0.0001	< 0.0001	180.60
		Fumarate	0.1628	< 0.0001	< 0.0001	133.56
		Malate	0.0376	< 0.0001	< 0.0001	139.16
Small organic acids	A0080	2-Hydroxy-3-methylbutyrate	0.6120	0.0394	0.0391	122.2
	A0405	Glutarate	0.5130	0.0191	0.0259	113.3
Amino acids and derivatives	A0008	Alanine	0.0010	0.0078	0.0282	69.5
	A0051	Sarcosine	0.4840	0.0028	0.0043	144.9
	A0181	Serine	0.1600	0.0048	0.0053	177.4
	A0202	Ethanolamine	0.9920	0.0078	0.0018	117.9
	A0280	Cysteamine	0.6670	< 0.0001	0.0002	130.2
	A0434	Aspartic acid	0.2190	0.0385	0.0391	119.2
	A0502	Aminomalonate	< 0. 0001	0.0158	0.02	117.6
	A0613	Creatinine	0.2010	0.0372	0.0361	131.2
	A0622	Cysteine	0.0020	0.0006	0.0004	117.3
	A0702	2-Aminoadipic acid	0.0110	0.0376	0.0424	73.3
	A0813	Glutamine	0.7640	0.0186	0.0238	119.9
	A0820	O-Phosphorylethanolamine	0.1160	< 0.0001	< 0.0001	132.6
Lipid metabolism	A0199	Octanoic acid	< 0.0001	0.0379	0.1511	80.8
	A0328	Nonanoic acid	0.6900	0.0365	0.0361	86.7
	A0482	Decanoic acid	< 0.0001	0.0063	0.0105	72.1
	A0720	Dodecanoic acid	0.2730	0.0323	0.0496	81.8
	A0991	Octadecan	0.1560	0.0409	0.0306	111.9
	A1087	1-Monopalmitin	0.9430	0.0006	0.0014	138.1
	A1092	1-Monolinolein	< 0. 0001	0.004	0.0361	164.5
	A1093	2-Oleoylglycerol	0.7410	0.0053	0.0053	177.4
	A1099	Fatty acid amide	< 0.0001	0.0042	0.0039	55.1
Nucleotide metabolism	A0294	Uracil	< 0.0001	< 0.0001	< 0.0001	778.4
	A0554	6-Azathymine	0.9640	0.0909	0.0458	113.2
	A0836	Hypoxanthine	0.0110	< 0.0001	< 0.0001	256.3
	A1086	Inosine	< 0.0001	< 0.0001	< 0.0001	1781.3
	A1105	2-Hydroxypyrimidine	0.8600	0.0593	0.0218	139.6

The values in the last column represent the relative molecular abundance in CO₂-stunned animals, expressed as a percentage in relation to electrically stunned animals (set to 100%). Values greater than 100% indicate an increase in metabolite levels under CO₂ stunning. Significant differences were calculated using ANOVA (for parametric data) and the Wilcoxon test (for non-parametric data).

### Differential energy demand and TCA cycle alterations in CO₂- versus electrically-stunned pigs

**Fig. 2:**
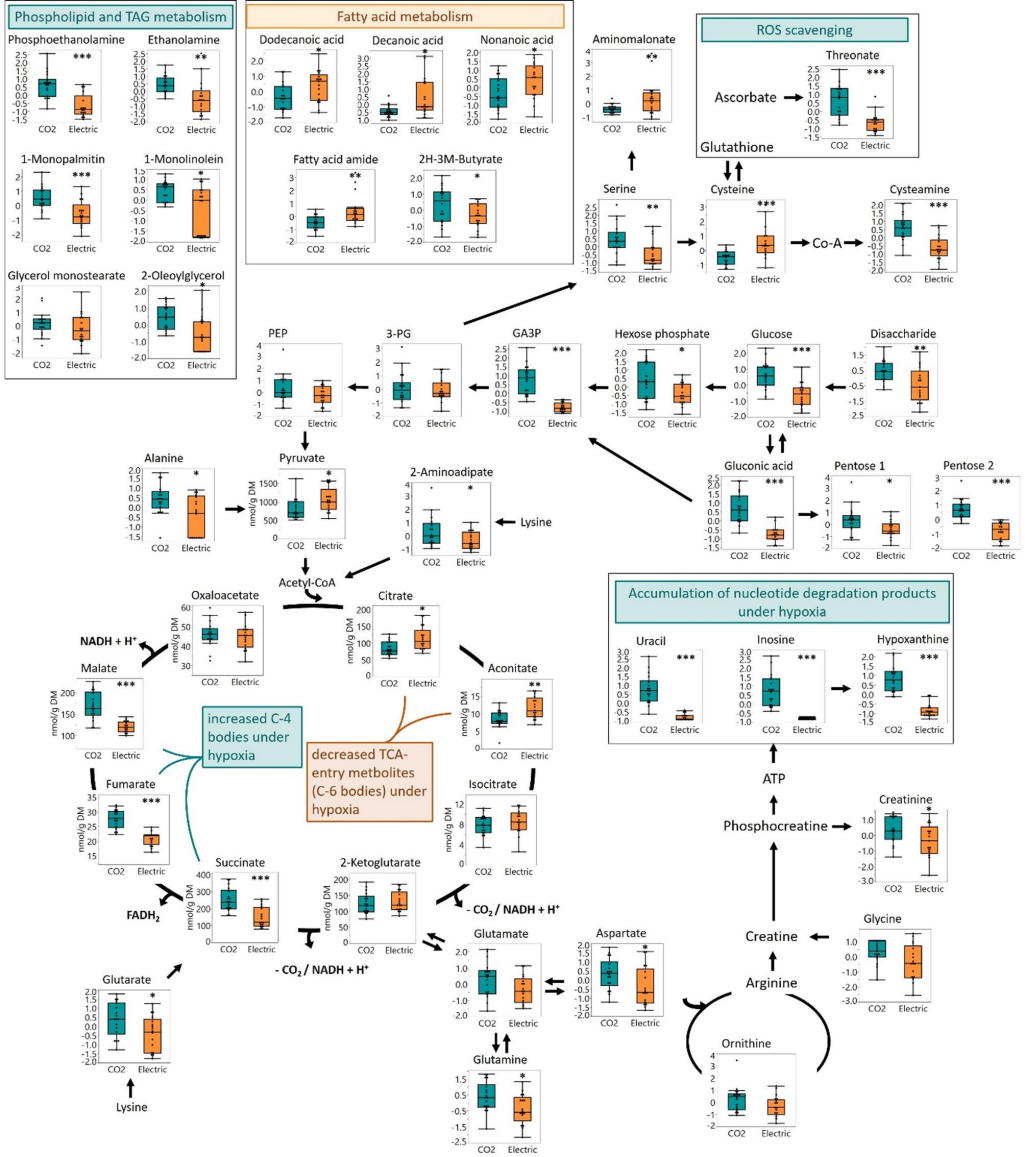
Illustration of metabolites detected by GC–MS metabolite analysis, which were affected by the stunning methods, in context of their pathways or classes. Significance levels are: ≤ 0.05: *; ≤ 0.01: **; ≤ 0.001: ***. Whiskers in Box plots represent min and max values.

Under CO₂ stunning, the most notable findings were the substantial accumulation of ATP degradation products, including inosine and hypoxanthine, as well as uracil, a breakdown product of UDP-sugars (Fig. 2, Table 1). These results were accompanied by differences in the energy-producing pathways of glycolysis and the TCA cycle.

In the energy consuming part of glycolysis, up to the cleavage into glyceraldehyde-3-phosphate (GA3P), metabolite levels were elevated under CO_2_ conditions compared to electrical stunning. However, no significant differences were observed in the energy-generating part of glycolysis, nor in lactic acid levels, between the two stunning techniques. Upon entry into the TCA cycle, a shift between the two stunning methods was again observed. Citrate and aconitate concentrations were significantly lower under CO₂ stunning, while following decarboxylation from C6 to C4 acids, such as succinate, fumarate, and malate, the concentrations were higher under CO₂ conditions (Fig. 2).

Further analysis of purines by LC–MS was conducted to assess the concentrations of key energy substrates (Table 2). No significant difference in ATP levels was observed, but notable differences were found in other adenylates. ADP levels were slightly higher, while AMP and adenosine concentrations were significantly elevated in blood from electrically stunned pigs. In contrast, adenine levels were significantly elevated in blood samples from CO₂-stunned pigs, whereas no differences were detected for the degradation product IMP. However, further breakdown into inosine and hypoxanthine revealed substantial differences between the two stunning methods, with significantly greater accumulation observed following CO₂ stunning. These findings were consistent with the patterns of inosine and hypoxanthine detected in GCxGC-qMS profiling. From guanylate-derived purines, guanosine and guanine were detected. Guanosine was only detected in blood from CO_2_-stunned pigs, while guanine showed a slight difference between the two techniques. Overall, the total blood purine concentration was markedly increased following CO_2_ stunning.

**Table 2:** Concentrations of purines in blood after electrical and CO_2_ stunning.

Compound	Treatment	Mean (nmol/g)	Std. dev	*p* value
ATP	CO_2_	17.26	4.59	0.4061
	electric	15.77	4.41	
ADP	CO_2_	35.98	5.59	0.0496
	electric	40.90	8.82	
AMP	CO_2_	21.68	8.11	0.0001
	electric	54.86	32.87	
Adenosine	CO_2_	0.483	0.91	0.0017
	electric	0.811	0.50	
Adenine	CO_2_	2.311	0.53	< 0.0001
	electric	1.273	0.32	
IMP	CO_2_	22.12	3.44	0.0759
	electric	24.60	4.04	
Inosine	CO_2_	118.16	42.14	< 0.0001
	electric	13.21	4.81	
Hypoxanthine	CO_2_	206.08	40.62	< 0.0001
	electric	100.89	21.70	
Guanosine	CO_2_	9.01	2.60	< 0.0001
	electric	n.d	n.d	
Guanine	CO_2_	8.28	1.57	0.0496
	electric	7.01	1.78	
Sum	CO_2_	441.35	85.46	< 0.0001
	electric	260.31	61.87	

### Cellular and oxidative stress markers following CO₂ and electrical stunning

Notably, the ascorbate degradation product threonate was more abundant in blood from CO_2_-stunned pigs compared to electrically stunned pigs. Among the glutathione precursors, only cysteine showed a significant difference, with higher levels observed after electrical stunning (Table 1).

To draw direct conclusions about stress response induction under such acute physiological conditions, we analyzed the mRNA expression of heat shock proteins HSP27, HSP70, HSP90, and CRYAB. However, no differences in expression were detected between the two stunning methods.

### Limited impact on proteinogenic amino acids

The profile of proteinogenic amino acids was generally only slightly affected by the stunning method. Significant differences were observed for alanine, serine, aspartate and glutamine, which showed slightly higher levels after CO_2_-stunning, whereas cysteine was more abundant in blood from electrically stunned pigs (Table 1). Cysteamine, an intermediate in taurine biosynthesis from cysteine and a degradation product of Coenzyme-A, was found at significantly higher levels under CO_2_ conditions. 2-Hydroxy-3-methylbutyric acid, a conversion product of branched-chain amino acids, slightly increased under hypoxia, as did aminoadipate, a degradation product of lysine. In contrast, aminomalonate, which can arise from perturbed serine or threonine metabolism, accumulated more after electrical stunning (Table 1).

### Effects on lipid metabolism

Several blood lipids exhibited differences between the two stunning techniques. Among these, the monoacylglycerides 1-monopalmitin, 1-monolinolein and 2-oleoylglycerol were significantly more abundant following CO_2_ treatment (Table 1). Additionally, the phospholipid precursors O-phosphorylanolamine and ethanolamine displayed the same trend. In contrast, medium-chain fatty acids, specifically octanoic, nonanoic, and decanoic acids, as well as the long-chain fatty acid dodecanoic acid, were more abundant in blood after electrical stunning (Table 1).

## Discussion

This study investigated how different stunning methods affect blood metabolite profiles, with the aim of identifying associated physiological pathways and their relevance to *post mortem* development. Both stunning methods cause significant physiological stress and place a heavy burden on energy metabolism due to the procedures involved in inducing unconsciousness prior to exsanguination. Electrical stunning triggers generalized seizures through neuronal depolarization, increasing muscular activity and oxygen demand. In contrast, exposure to high levels of CO₂ causes respiratory acidosis due to hypercapnia, accompanied by hypoxia. The present study investigated whether, given their different modes of action, different metabolic responses are elicited.

### Distinct metabolic profiles induced by the stunning method

**Fig. 3:**
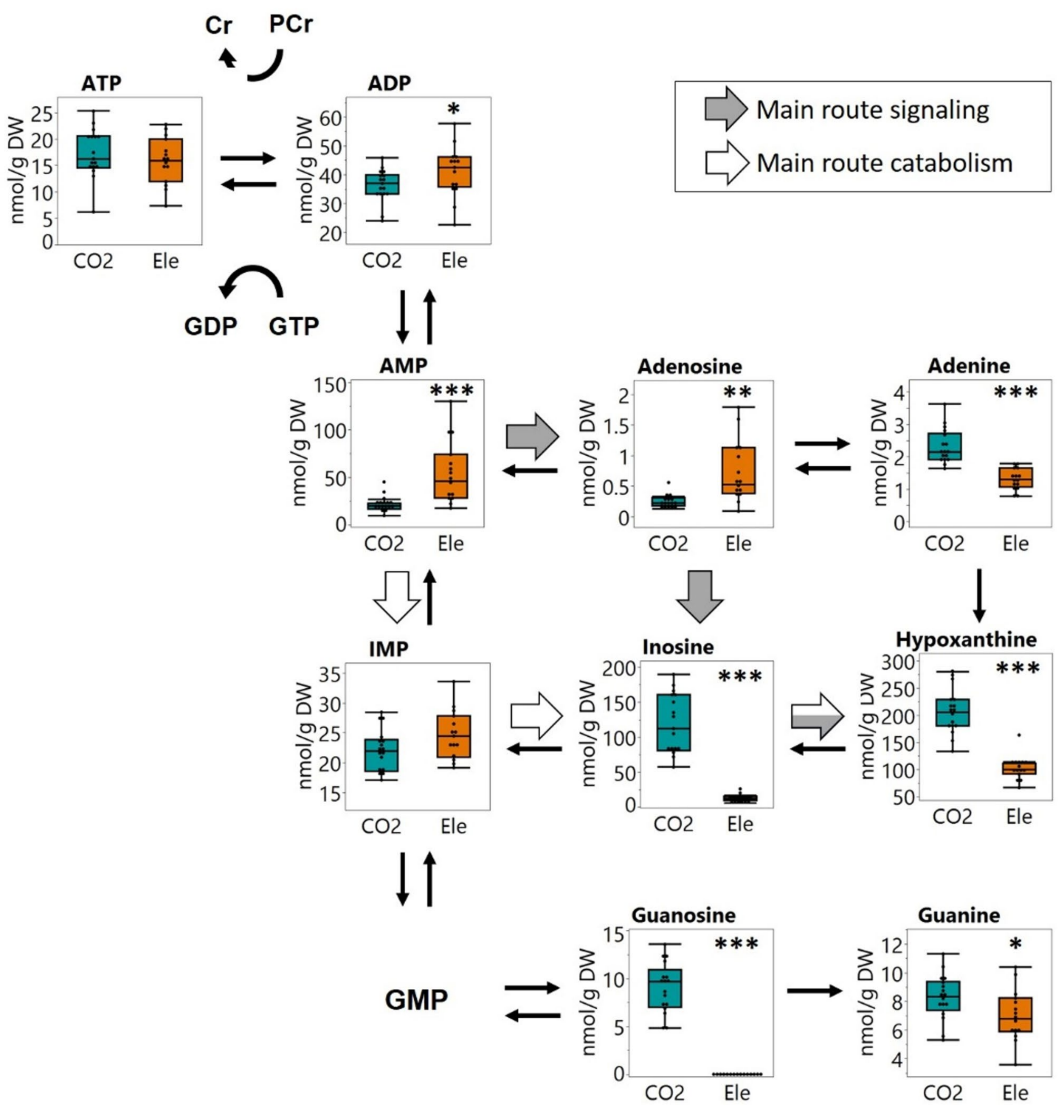
Detected purine metabolites and indicated catabolic routes for purinergic signalling and energy metabolism. Significance levels are: ≤ 0.05: *; ≤ 0.001: ***. Whiskers in Box plots represent min and max values.

Indeed, the different metabolic responses of the pigs to the two stunning techniques were clearly reflected in their overall metabolite profiles (Fig. 1). Despite considerable individual variability—likely due to physiological and behavioral differences—a clear separation between the two groups was evident in the PCA of all detected metabolic features (Fig. 1), as well as in targeted analyses of purines and TCA cycle metabolites (Figs. 2 and 3). The pronounced effects of the stunning methods on blood metabolites indicate that specific interventions can significantly alter metabolic patterns within seconds.

### The stunning technique has a decisive influence on the processes of energy metabolism

Several aspects of central metabolism were differentially impacted during the brief period of stunning and subsequent bleeding. Most notably, the stunning techniques resulted in pronounced differences of energy metabolites. Stunning imposes an enormous burden for energy metabolism per se. The significant differences in inosine and hypoxanthine clearly indicated a strong impact of CO_2_ stunning on adenosine phosphate degradation and regeneration (Fig. 3). This can be attributed to the sudden drop in oxygen and pH as well as the rise in pCO_2_ under gas stunning, which disrupts ATP production via the mitochondrial electron transport chain (ETC)^20–24^. As ATP turnover far exceeds its cellular concentration, supply and demand must be tightly balanced^25^. Thus, stabilization of the ATP supply for energy-consuming processes is existential, and different metabolic strategies exist to overcome acute ATP shortages: phosphocreatine utilization (phosphagen system), anaerobic glycolysis, interconversion of purine phosphates and substrate-level phosphorylation. Among these, the phosphagen system provides the most rapidly accessible energy source. The critical role of phosphocreatine in cell survival under anoxic or severely hypoxic conditions has been highlighted in several studies^26,27^. In this direction, Braasch^28^ firstly reported significant depletion of PCr and ATP within just 30 s of coronary occlusion in the ischemic area. Similarly, Norberg^29^ observed a marked decline in PCr levels in the cerebral cortex within 10–20 s of hypoxia. Our study suggests a similar scenario in the blood of CO_2_-stunned pigs where elevated creatinine levels indicated a quick shift from ATP production via the ETC to phosphocreatine utilization (Fig. 2). This is also consistent with Wilken^26^, who reported an almost complete depletion of PCr and a 54% reduction in ATP levels after 30 min of anoxia in mouse pups. Similarly, Tsuji et al.^30^ documented a significant drop in PCr in the brain within just 3–4 min of exposure to only 4% oxygen. Although the cited studies reflect only hypoxic or ischemic conditions and not the specific effects of hypercapnia and acidosis, the rapid depletion of PCr seems to represent a shared metabolic response to impaired oxidative phosphorylation.

ATP can also be regenerated via anaerobic glycolysis, which is strongly upregulated under oxygen-limited conditions. This is reflected in rapid glycogen depletion during ischemia^31^ and increased lactic acid production during intense physical activity^32^ or epileptic seizures^4,33,34^. However, the metabolite levels associated with anaerobic glycolysis (e.g., pyruvate, lactic acid) did not differ between the two stunning techniques. This is likely due to the fact that both mechanisms independently promote lactate formation.

### CO₂ stunning triggers elevated purine catabolites compared to electrical stunning

While immediate energy demands are met by the phosphagen system, additional energy supply can be facilitated through increased activity of the adenylate kinase (AK), which catalyzes the reaction 2ADP—> ATP + AMP. AMP is subsequently deaminated to IMP to further favor the AK reaction, and IMP might in a following step be degraded to inosine and hypoxanthine^35^. Under hypoxic conditions, either caused by ischemia or elevated muscle activity, increased AK activity along with higher AMP levels has been observed, and related to cellular metabolic adaptations^36–38^. The lower AMP amounts observed under CO₂ stunning conditions might be explained by its rapid degradation, as indicated by the substantial accumulation of inosine and hypoxanthine in comparison to electrical stunning. Such rapid accumulation of purine degradation products in blood serum has been reported under hypoxic conditions following various clinical syndromes^31,39,40^ and suggested as an indicator for ATP depletion^41^. As a consequence, hypoxanthine and inosine have been proposed as biomarkers for cardiac ischemia^42^.

However, most medical studies have focused on time frames spanning several minutes–hours. In contrast, our findings suggest a very rapid accumulation of inosine and hypoxanthine upon extreme conditions—hypercapnia and severe hypoxia occurring within just a few seconds. An interesting assumption was presented in a study by Miller et al.^43^, who analyzed the metabolic responses of rat dentate granule neurons during acute electrical stimulation: An increase in exogeneous inosine has been linked to fuel glycolysis via the nonoxidative pentose phosphate pathway (PPP) by using the ribose from inosine as a substrate for generating G6P. While this hypothesis is not directly associated with CO_2_ stunning, the elevated levels of inosine, hypoxanthine and hexose phosphate (Fig. 3) observed in our study could also suggest a similar mechanism for nonoxidative energy generation. In addition, increased purine metabolites levels have been observed in mouse blood following seizures, and were correlated with seizure severity and brain damage^44^. In our findings, the total purine concentration after CO_2_ stunning exceeded the level after electrical stunning (Table 2), potentially indicating a more severe reaction following anaerobia and acidosis.

In addition to their role as energy equivalents, extracellular ATP and its intermediates are important signaling molecules within the purinergic signaling cascade^45^. A range of selective receptors have been identified that mediate tissue-specific adaptive responses to maintain purine-dependent homeostasis^46–49^. In response to acute hypoxia, ATP is rapidly exported and inactivated to adenosine in the vasculature, which is further inactivated through deamination to inosine and hypoxanthine. It is therefore tempting to speculate that the substantial differences in inosine and hypoxanthine levels detected herein—markedly higher after CO_2_ stunning—may be a consequence of both extracellular signaling, where adenosine is degraded, and energy production and sensing through the orchestrated activities of AK, AMPD and AMPK, and further IMP degradation^35,36,50^. The large difference in total purine concentration in blood suggests that, responses upon CO_2_ stunning conditions occur much faster than under electrical stunning. The rapid excretion of purines into the bloodstream may serve as a fast alarm signal for energy imbalance and adaption.

### Impaired glycolytic flux during CO₂ stunning

The rate of glycolysis is strongly influenced by pH changes, primarily due to the sensitivity of phosphofructokinase (PFK), a major regulatory enzyme, to acidification. Under CO₂ stunning, the associated rapid drop in pH likely impairs key enzymatic steps, with inhibition of PFK contributing to the accumulation of upstream glycolytic intermediates. Foldbergrova^51^ demonstrated in rat brain tissue that rising CO₂ concentrations (6–40%) led to increased glucose and glucose-6-phosphate levels, consistent with impaired glycolytic flux due to intracellular acidification. LaMonte et al.^52^ similarly emphasized the high sensitivity of glucose metabolism to pH changes. Spriet et al.^53^ demonstrated in vitro that acidosis significantly reduces PFK activity during short-term muscle contractions, highlighting the direct inhibitory effect of low pH on glycolysis. However, they caution that in vivo conditions are more complex, and the extrapolation of in vitro findings to intact muscle metabolism requires careful consideration of additional regulatory factors. Accordingly, the elevated glucose and hexose phosphate levels observed in CO₂-stunned pigs likely reflect this complex, pH-dependent inhibition.

Compared to CO₂ stunning, electrical stunning showed a greater consumption of C6 sugars. This difference abruptly stopped between the oxidation and reduction reactions from glycerinealdehyde-3-phsohate (GA3P) to 3-phosphoglycerate (3PG), suggesting a metabolic bottleneck at this NAD⁺-dependent step. Apart from red blood cells, the abrupt disruption of the mitochondrial ETC during CO₂ stunning prevents NAD⁺ regeneration, reducing the NAD⁺/NADH + H⁺ ratio and potentially explaining the observed GA3P accumulation. Elevated hexose phosphate levels may also result from metabolic tailback or feedback inhibition. Additionally, the higher glucose levels in CO₂-stunned pigs could also result from imbalances in adenosine phosphate pools, as two ATP molecules are consumed in the initial steps of glycolysis. Limited ATP availability may therefore further impair glucose phosphorylation and utilization.

### Disrupted TCA cycle dynamics and C4 acid accumulation under CO₂ stunning

In the initial steps of the TCA cycle, electrically stunned pigs showed higher levels of citrate and aconitate, while similar amounts of 2-ketoglutarate were detected in both groups. In contrast, after the second decarboxylation step, CO_2_-stunned pigs exhibited a significantly higher abundance of C4 organic acids. This pattern suggests a reduction in TCA cycle flux under CO_2_ conditions, consistent with known adaptive responses to hypoxia^54,55^. Norberg^56^, by contrast, reported a time-dependent increase in the TCA metabolite pool over 1–30 min at moderately reduced oxygen levels (6–8%). However, most of these studies focus on slower responses to hypoxia and do not reflect the acute and extreme conditions during stunning—particularly the combined effects of hypercapnia and a rapid pH drop. The precise metabolic consequences of this acidification remain poorly understood.

The elevated C4 acids may result from disrupted NADH and FADH₂ oxidation due to rapid electron transport chain (ETC) failure. Limited NAD⁺ availability likely impairs mitochondrial malate dehydrogenase, leading to malate accumulation and potentially driving the reverse conversion of oxaloacetate to malate^57^, thereby supporting NAD⁺ regeneration. This enables continued function of the α-ketoglutarate dehydrogenase complex (KGDHC), which drives substrate-level phosphorylation via the conversion of succinyl-CoA to succinate, coupled with GDP to GTP conversion and ATP generation. This bypass pathway has been reported to be upregulated under hypoxic conditions^57–59^ and is regulated by ADP, ATP, pH, and Ca^2^⁺^59,60^. Reduced oxaloacetate availability for condensation with acetyl-CoA may contribute to the observed decreases in citrate and aconitate levels^61^. Additionally, ETC dysfunction may impair the malate-aspartate shuttle, which requires NAD^+^ for the oxidation of malate to oxaloacetate in the mitochondrial matrix. As NAD⁺ becomes limiting, malate may accumulate further. The observed increase in fumarate levels could reflect a backflux reaction, shifting the succinate-malate equilibrium in response to rising malate concentrations.

### Amino acids related to the TCA cycle and oxidative stress regulation

The central amino acids glutamine (Gln), aspartic acid (Asp) and alanine (Ala) were slightly more abundant in the blood of CO_2_-stunned pigs. The observed higher levels of Ala and Asp might be due to their role as products of transaminase reactions yielding ɑ-ketoglutarate (ɑKG) for substrate-level phosphorylation provision. This assumption is supported by data from hypoxia studies^56,57,62^. Levels of Gln were higher in CO_2_-stunned pigs than in electrically stunned pigs, indicating that CO_2_-induced hypoxia and hypercapnia during pig stunning may trigger additional reactions —likely pH-dependent, compared to the hypoxic conditions applied during medical studies. In this context, LaMonte^52^ detected a stronger glutamate labeling under acidosis in their ^13^C glucose tracer flux measurements.

Amino acids cysteine, cysteamine, serine and aminomalonate were also differentially affected. Aminomalonate, which is involved in calcium-binding activity of proteins and has been identified as a toxicity marker in previous studies^63^, was more accumulated after electrical stunning compared to CO_2_ stunning. The origin of aminomalonate is not fully understood, and Copley^64^ suggested that it might arise as a constituent of proteins originating either from misincorporation during protein synthesis or as a result of posttranslational modification from another amino acid. It has also been hypothesized that it might be generated by the oxidation of serine via enzymatic or radical-mediated processes.

Electric shock and acidosis have been related to the generation of ROS^52,65^. A greater turnover of ROS-scavenging compounds can be suspected in electrically stunned pigs. Cysteine accumulation in these animals may result from glutathione (GSH) degradation by membrane-bound gamma-glutamyl transpeptidase (GGT) and dipeptidases^66,67^. In addition, GSH de novo synthesis has been found to be reduced under acidosis^52^. Further evidence of elevated ROS levels in electrically stunned pigs comes from higher threonate levels—a degradation product of ascorbate and dehydro-l-ascorbic acid (DHA)^68^. These findings collectively suggest that electrical stunning induces slightly higher oxidative stress compared to CO_2_ stunning.

On the other hand, cysteamine, a product of the constitutive degradation of coenzyme A, also functions as an antioxidant. However, due to its high reactivity and broader role in sulfur metabolism, including taurine production, the higher levels observed after CO_2_ stunning cannot be definitively linked to counteracting oxidative stress.

### Fatty acid chain length differences

The higher abundance of medium-chain fatty acids observed after electrical stunning is in accordance with the finding that fatty acid oxidation depends on O_2_ availability. Achten et al.^69^ reported a strong correlation between the maximal rate of fat oxidation and VO_2_ max. Comparing the acute anoxia during CO_2_ stunning with the presumably less severe O_2_ shortage during electrical stunning it can be concluded that ß-oxidation of fatty acids is utilized to fill the acetyl-CoA pool and fuel the TCA cycle. This interpretation aligns with the higher citrate levels observed after electrical stunning. At the moment of death, these ongoing oxidation processes were interrupted by the bleeding of the animals. Furthermore, lower amounts of MAGs in electrically stunned pigs may also reflect enhanced fat hydrolysis, which supplies free fatty acids for oxidation under these conditions.

### Associations to early post mortem processes

Both stunning techniques resulted in distinct metabolic conditions at the time of death and are therefore likely to influence *post mortem* biochemical processes. Previous studies on carcass and meat quality have indicated better carcass characteristics of CO_2_-stunned pigs^70,71^. However, regarding meat quality, particularly color, water-holding capacity, and tenderness, the literature presents inconsistent findings^10,71–73^. Given that glycolysis and ATP degradation play central roles in early *post mortem* muscle metabolism, the observed differences in energy-related metabolites in this study are likely to affect resulting meat quality. Interestingly, the faster degradation of purines observed after CO₂ stunning could be interpreted as a sign of greater metabolic stress, suggesting a potential advantage for electrical stunning. Conversely, the higher levels of glycolytic intermediates detected under CO₂ conditions may support a more favorable energy status, which in turn could benefit meat quality. This apparent contradiction mirrors the divergent findings in the literature and underscores the need for a comprehensive evaluation of all slaughter-related factors. As meat quality depends also on pre-slaughter stress, a holistic approach that accounts for the entire pre- and perimortem context is essential to reliably assess the impact of stunning methods on meat quality.

## Conclusions

Metabolite profiling has proven to be a valuable tool for assessing physiological responses associated with different stunning methods. In this study, both CO₂ and electrical stunning resulted in profound metabolic alterations in blood at death, albeit via distinct biochemical pathways. CO₂ stunning led to strong purine degradation, with significantly increased inosine and hypoxanthine levels (both *p* < 0.0001), and accumulation of TCA cycle intermediates (succinate, fumarate, malate; all *p* < 0.0001), suggesting mitochondrial dysfunction and purinergic emergency signaling. In contrast, electrical stunning showed relatively higher levels of glycolytic turnover, reflected by reduced levels of glucose (*p* < 0.0001) and elevated TCA cycle entry metabolites including citrate (*p* = 0.0053) and aconitate (*p* = 0.0009). These differences likely reflect distinct physiological stress responses: CO_2_-induced acidosis may impair glycolysis, while electrical stunning causes less severe hypoxia. Furthermore, differences in NAD⁺/NADH + H⁺ redox balances between stunning methods may affect both glycolysis and TCA cycle activity, contributing to the observed metabolite patterns.

Given the substantial impact of energy metabolism on meat quality development and its dependence on the stunning method, correlating blood metabolite profiles directly with meat quality appears promising. However, it is well known that preslaughter processes also significantly affect meat quality. Therefore, future studies should integrate not only *post mortem* analyses but also data from preslaughter handling and prestunning blood profiles. Since gas stunning remains the most widely used method in high-throughput slaughterhouses, further studies should investigate how variations in gas composition and pre-slaughter animal handling affect both biochemical processes and stress responses.

## Electronic supplementary material

Below is the link to the electronic supplementary material.

Supplementary Material 1

Supplementary Material 2

Supplementary Material 3

## Data Availability

All data generated and analysed during this study are included in this published article and its supplementary information files. The raw datasets (in the vendors file format) are available from the corresponding author on reasonable request.

## Abbreviations

AMP: Adenosine 5′-monophosphate
ADP: Adenosine-5′-diphsphate
ATP: Adenosine-5′-triphsphate
GMP: Guanosine-5-monophosphate
CO_2_: Carbon dioxide
TCA: Tricarboxylic acid cycle
ROS: Reactive oxygen species
HIF: Hypoxia-inducible factor
GC: Gas chromatograph/gas chromatography
qMS: Quadrupol mass spectrometer
QC: Quality check
MSTFA: *N*-methyl-*N*-(trimethylsilyl)trifluoroacetamide
TMCS: Trimethylchlorosilane
MAH: O-methoxylamine hydrochloride
NaOH: Natrium hydroxide
MTBSTFA: *N*-methyl-*N*-(tert-butyldimethylsilyl)trifluoroacetamide
TBDMCS: Tert-butyl dimethyl chlorsilan
NaBD4: Natrium borodeuteride
HCl: Hydrogen chloride
LC: Liquid chromatography
ESI: Electrospray ionization
QToF: Quadrupol time of flight mass analyzer
MS: Mass spectrometer/mass spectrometry
PCA: Principal component analysis
OPLS-DA: Orthogonal projection to latent structures discriminant analysis
ANOVA: Analysis of variance
VIP: Variable importance projection
UDP: Uridine diphosphate
GA3P: Glyceraldehyde 3-phosphate
C4, C6: Four and six carbon bodies
mRNA: Messenger ribonucleic acid
HSP: Heat shock protein
CRYAB: Crystallin alpha B
ETC: Electron transport chain
PCr: Phosphocreatin/creatine phosphate
O2: Oxygen
NAD+: Nicotinamide adenine dinucleotide (oxidized form)
AK: Adenylate kinase
AMPK: Adenosine monophosphate activated protein kinase
PPP: Pentose phosphate pathway
AMPD: Adenosine monophosphate deaminase
3PG: 3-Phosphoglycerate
NADH: Nicotinamide adenine dinucleotide (reduced form)
OAA: Oxalo acetic acid
FADH2: Flavin adenine dinukleotide (reduced form)
FAD: Flavin adenine dinukleotide (oxidized form)
KGDHC: Alpha-ketoglutarate dehydrogenase complex
Ca: Calcium
GGT: Gamma-glutamyl transpeptidase
GSH: Glutathione
DHA: Dehydro-l-ascorbic acid
MAG: Monoacylglycerol
